# Adrenal Insufficiency Due to Bilateral Adrenal Non-Hodgkin's Lymphoma: A Case Report

**DOI:** 10.7759/cureus.7359

**Published:** 2020-03-22

**Authors:** Fahed S Bangash

**Affiliations:** 1 Internal Medicine, Hull University Teaching Hospitals NHS Trust, Hull, GBR

**Keywords:** adrenal insufficiency, diffuse large b cell lymphoma, primary adrenal insufficiency, addison's disease, non hodgkin's lymphoma, positron emission tomography-computed tomography

## Abstract

Primary adrenal insufficiency is a rare condition due to the impairment of adrenal glands. Previously, tuberculosis damaging adrenal glands was attributed as the main cause for it; whereas, nowadays autoimmune disease is the most common cause of it. However, rarely metastatic malignancy can cause adrenal insufficiency as well. This is a case report of a 72-year-old who presented with a three-month history of being generally unwell. Investigations showed bilateral adrenal masses with a positive short synacthen test (SST) for adrenal insufficiency. Adrenal insufficiency was managed with hydrocortisone and fludrocortisone while the biopsy showed diffuse large B-cell lymphoma (DLBCL). He underwent positron emission tomography/computed tomography (PET/CT) which showed adrenal hypermetabolic disease with retroperitoneal involvement. He was treated as stage 4 non-Hodgkin's lymphoma (NHL) with two cycles of rituximab, cyclophosphamide, doxorubicin, vincristine, and prednisone (R-CHOP) chemotherapy on the same admission; he was discharged home with further follow up for chemotherapy sessions. This case is unique as adrenal involvement in DBCL rarely leads to adrenal insufficiency; his symptoms resolved after receiving adrenal insufficiency treatment which further signifies the importance of its diagnosis and management.

## Introduction

Adrenal insufficiency is a rare disease with a prevalence of up to 114 people per million population [1-3]. Adrenal insufficiency is known to have associations with other conditions, therefore, knowledge of this can lead to prompt diagnosis and earlier management once this diagnosis is made. Lymphomas can cause adrenal insufficiency by involving adrenal glands, although the majority of them arise from lymph nodes, up to a quarter develop from extra-nodal sites [4]. Disseminated non-Hodgkin's lymphoma (NHL) can involve adrenal glands in up to 24% of patients as reported in an autopsy study [5].

Adrenal insufficiency is uncommon with adrenal involvement from malignancies since a small functioning reserve of the adrenal glands is enough to sustain life. In a previous study, only four cases were reported to have adrenal insufficiency out of 127 patients with NHL [6]. In this report, a rare case of adrenal insufficiency due to NHL is described, with symptoms overlapping with the underlying malignancy. The aim of the article is to highlight the importance of diagnosis and treatment of adrenal insufficiency, as in this case, where the patient's symptoms significantly improved after receiving treatment.

## Case presentation

A 72-year-old fully independent gentleman presented with a three months history of being generally unwell and lethargic with further deterioration of symptoms over the last few days. There was a past medical history of type 2 diabetes mellitus (T2DM) and hypertension (HTN). There was an undocumented history of weight loss along with the absence of cough, shortness of breath, lower urinary tract symptoms, fever, diarrhoea, or abdominal pain. He did not have any smoking or recent travel history.

Initially, he was admitted at a local hospital with observations showing blood pressure of 94/44 mmHg and a random blood sugar level of 4.8 mmol/L (4.0-5.4 mmol/L when fasting). His medications for HTN and T2DM were optimised in keeping with lower normal blood pressure and normal blood glucose readings. Blood tests showed hyponatremia with serum sodium of 128 mmol/L (136-142 mmol/L) and serum potassium of 5.1 mmol/L (3.5-5.0 mmol/L). In keeping with his symptoms, especially the history of weight loss, a computed tomography (CT) scan of the chest, thorax, abdomen, and pelvis was done to rule out any underlying malignancy. It showed bilateral adrenal masses with no other positive findings.

Short synacthen test (SST) was done due to his symptoms and findings of adrenal masses on the CT scan. It confirmed adrenal insufficiency for which he was started on oral hydrocortisone and fludrocortisone. His symptoms of fatigue and being generally unwell improved significantly. CT-guided adrenal gland biopsy was done which showed histological findings consistent with diffuse large B-cell lymphoma (DLBCL).

**Figure 1:**
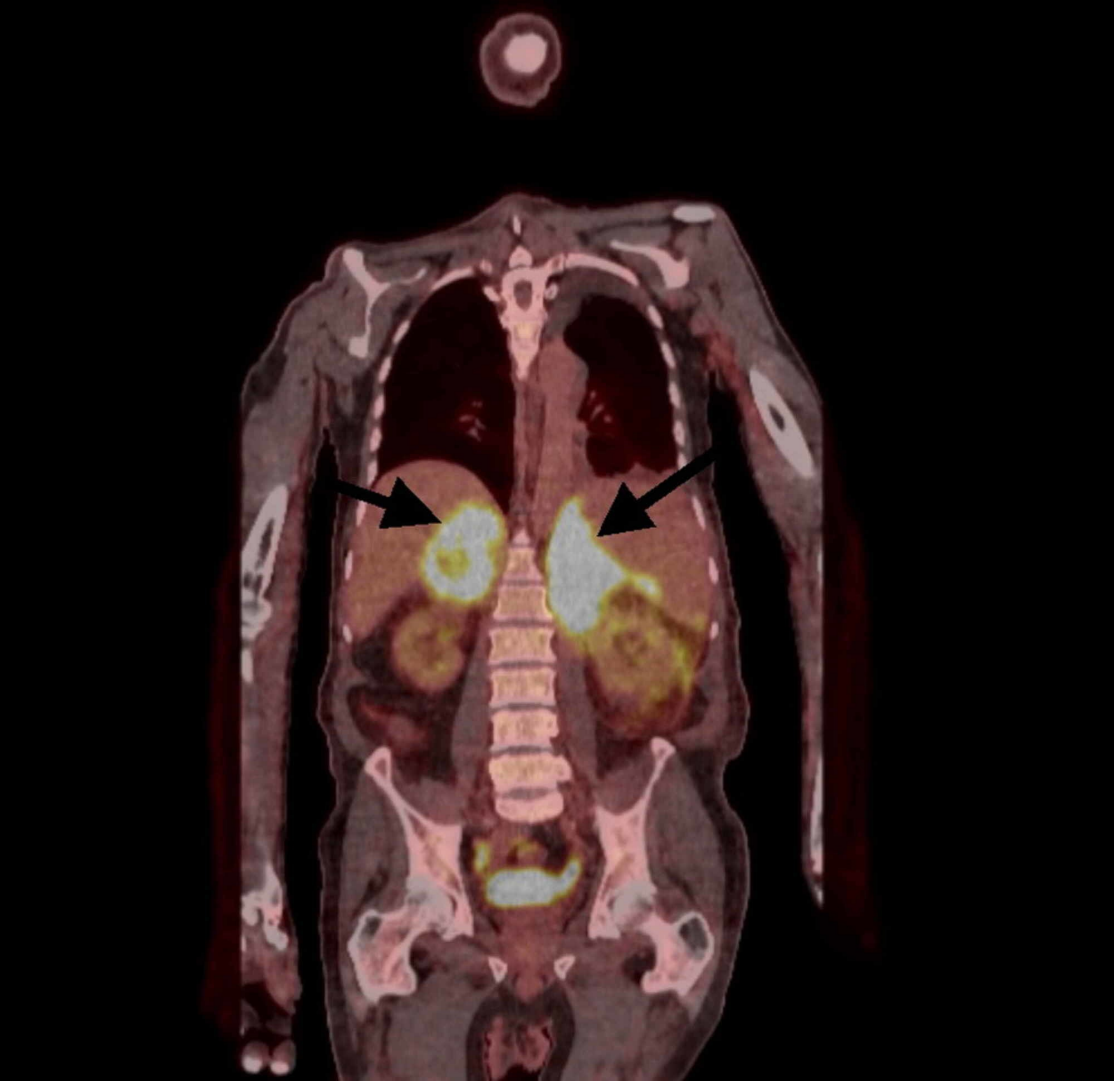
Positron emission tomography/computed tomography (PET/CT) coronal view Intensely hypermetabolic disease within the abdomen, involving both the adrenal glands, with conglomerate 18F-fluorodeoxyglucose (FDG) avid soft tissue extending inferiorly from the retroperitoneum and mesentery predominantly along the left side, extending for at least 21 cm craniocaudal standardized uptake value (SUV) max 15.4.

**Figure 2:**
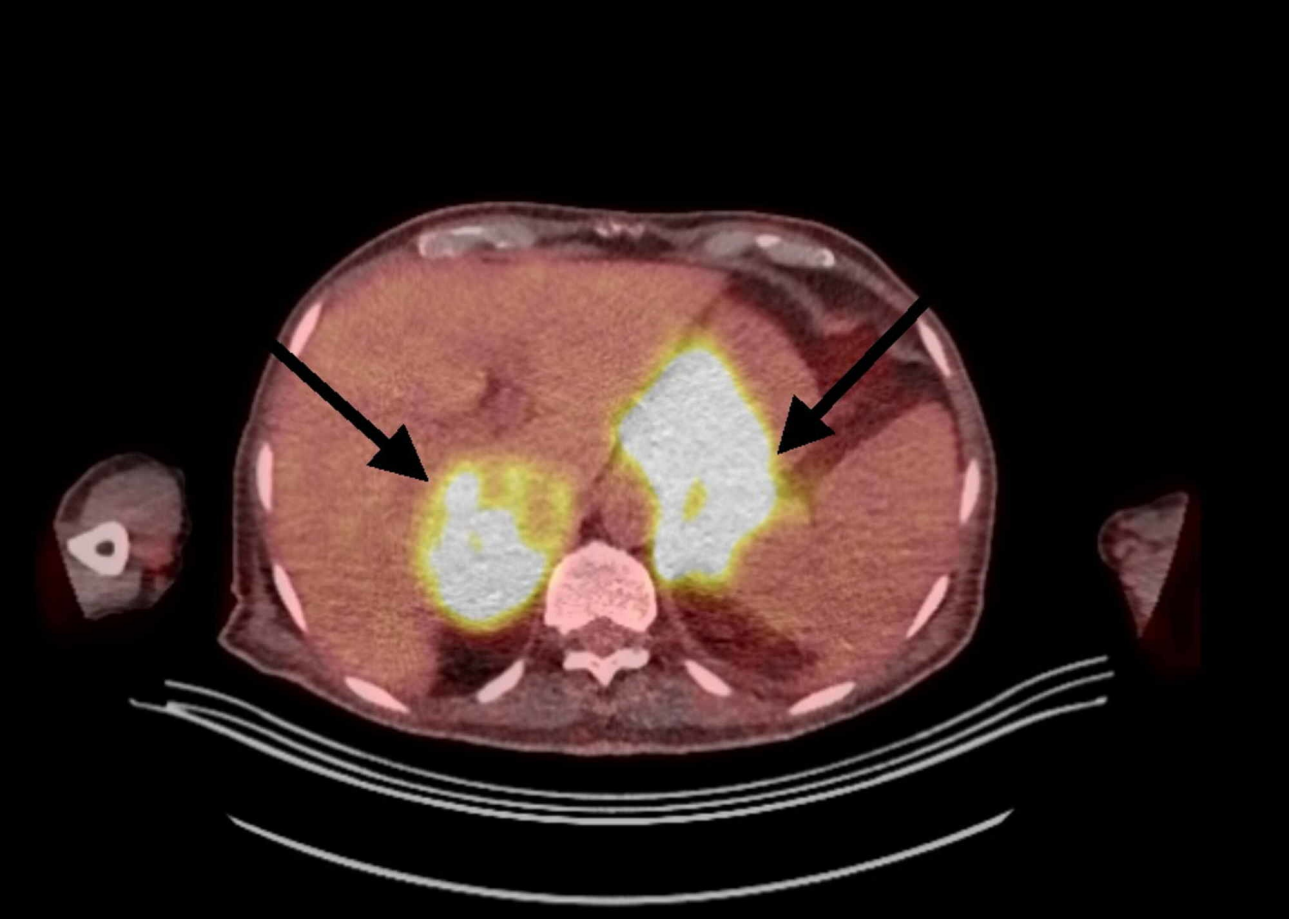
Positron emission tomography/computed tomography (PET/CT) transverse view Bilateral hypermetabolic adrenal glands; on the left side it is extending from the retroperitoneum and mesentery.

Moreover, he underwent positron emission tomography (PET) CT which showed intensely hypermetabolic disease within the abdomen, involving both the adrenal glands with conglomerate 18F-fluorodeoxyglucose (FDG) avid soft tissue extending inferiorly from the retroperitoneum and mesentery predominantly along the left side (Figures 1-2). There was also a 1.6 cm left posterior mediastinal node and standardized uptake value (SUV) max 6.4 with no other positive findings.

Bone marrow biopsy was done with no evidence of disease. He received high-dose steroids for four days for DLBCL followed by two cycles of rituximab, cyclophosphamide, doxorubicin, vincristine, and prednisone (R-CHOP) chemotherapy. During the inpatient stay, he was managed for neutropenic infection post-chemotherapy. He was discharged home on oral hydrocortisone and fludrocortisone after the resolution of neutropenic infection. He will be having regular follow up for monitoring and chemotherapy sessions.

## Discussion

Adrenal insufficiency has variable presentation varying from the spectrum of acute presentation of Addisonian crisis to the chronic presentation of vague symptoms. It is usually tricky to diagnose adrenal insufficiency as it is uncommon and many other conditions can present with the same symptoms, therefore, a low threshold of clinical suspicion is critical.

Malignancies are one of the known causes of involving adrenal glands due to the nature of its rich blood supply [6-7]. Histological diagnosis is very important as the differential diagnosis of bilateral adrenal masses can vary from benign adrenal tumour to tuberculosis, primary adrenal lymphoma, metastatic malignancy, and pheochromocytoma. Adrenal insufficiency due to metastasis to the adrenal glands is uncommon as up to 90% of the adrenal glands should be destroyed to cause adrenal insufficiency [8-9]. But there should be a low threshold for adrenal insufficiency and SST should be done in all patients with adrenal involvement as many symptoms of adrenal insufficiency can wrongly be attributed to the malignancy. 

As in the present case, the patient's chronic symptoms were likely due to a combination of metastatic disease and adrenal insufficiency but later it was most probably the reason he deteriorated and presented to the hospital, further evidenced by the resolution of his symptoms after being treated for adrenal insufficiency. On review of the literature and previous data of patients with symptoms predominantly due to underlying malignancy or adrenal insufficiency, the majority of the data on adrenal insufficiency along with adrenal metastasis is reported as case reports with a variable presentation [10-13]. A retrospective study of 30 years with 464 patients showed that only five patients had adrenal insufficiency [8]. This makes it difficult to differentiate the predominant factor causing symptoms but there is data mentioning the same point that managing adrenal insufficiency improves the quality of life [13].

## Conclusions

Patients with adrenal masses should be investigated with imaging and biopsy (if required) to establish a diagnosis. There should be a low threshold to test for adrenal insufficiency, especially in the background of malignancy with adrenal involvement, as symptoms may be wrongly attributed to the malignancy.
